# Cost-benefit analysis of vaccination: a comparative analysis of eight approaches for valuing changes to mortality and morbidity risks

**DOI:** 10.1186/s12916-018-1130-7

**Published:** 2018-09-05

**Authors:** Minah Park, Mark Jit, Joseph T. Wu

**Affiliations:** 1 WHO Collaborating Centre for Infectious Disease Epidemiology and Control, School of Public Health, Li Ka Shing Faculty of Medicine, The University of Hong Kong, G/F, Patrick Manson Building (North Wing), 7 Sassoon Road, Hong Kong SAR, People’s Republic of China; 2 Department of Infectious Disease Epidemiology, London School of Hygiene and Tropical Medicine, Keppel Street, London, WC1E 7HT UK; 3 Modelling and Economics Unit, Public Health England, 61 Colindale Avenue, London, NW9 5EQ UK

**Keywords:** Cost-benefit analysis, Economic evaluation, HPV, Vaccination

## Abstract

**Background:**

There is increasing interest in estimating the broader benefits of public health interventions beyond those captured in traditional cost-utility analyses. Cost-benefit analysis (CBA) in principle offers a way to capture such benefits, but a wide variety of methods have been used to monetise benefits in CBAs.

**Methods:**

To understand the implications of different CBA approaches for capturing and monetising benefits and their potential impact on public health decision-making, we conducted a CBA of human papillomavirus (HPV) vaccination in the United Kingdom using eight methods for monetising health and economic benefits, valuing productivity loss using either (1) the human capital or (2) the friction cost method, including the value of unpaid work in (3) human capital or (4) friction cost approaches, (5) adjusting for hard-to-fill vacancies in the labour market, (6) using the value of a statistical life, (7) monetising quality-adjusted life years and (8) including both productivity losses and monetised quality-adjusted life years. A previously described transmission dynamic model was used to project the impact of vaccination on cervical cancer outcomes. Probabilistic sensitivity analysis was conducted to capture uncertainty in epidemiologic and economic parameters.

**Results:**

Total benefits of vaccination varied by more than 20-fold (£0.6–12.4 billion) across the approaches. The threshold vaccine cost (maximum vaccine cost at which HPV vaccination has a benefit-to-cost ratio above one) ranged from £69 (95% CI £56–£84) to £1417 (£1291–£1541).

**Conclusions:**

Applying different approaches to monetise benefits in CBA can lead to widely varying outcomes on public health interventions such as vaccination. Use of CBA to inform priority setting in public health will require greater convergence around appropriate methodology to achieve consistency and comparability across different studies.

**Electronic supplementary material:**

The online version of this article (10.1186/s12916-018-1130-7) contains supplementary material, which is available to authorized users.

## Background

Health economic evaluations are used to inform medical procurement and reimbursement decisions by public and private healthcare providers. The most popular form of health economic evaluation is cost-effectiveness analysis (CEA), which often presents the ratio of the incremental cost of an intervention (from the perspective of either the healthcare provider or society) to the incremental health benefits of an intervention. A review conducted for the Bill & Melinda Gates Foundation of health economic evaluations of interventions related to malaria, tuberculosis, HIV/AIDS and vaccination in low- and middle-income countries found that, of 204 studies published in 2000–2013, 202 (99%) were CEAs [1].

Economic evaluations of large public health interventions such as new vaccination programmes attract particularly intense debates because of the high absolute costs (and potentially large benefits) involved [2]. A major focus of such debates has been about whether current economic evaluation techniques capture the full scope and value of these public health programmes. For instance, several reviews have found that vaccines may have broad, long-term societal consequences that are not always captured in CEAs [3, 4], although many of these benefits can, in principle, be monetised and included in CEA based on a broader societal perspective as recommended by the US Second Panel on Cost-Effectiveness in Health and Medicine [5, 6]. Such broader, non-health benefits of an intervention include effects on future productivity and consumption, social services, educational achievement and other societal impacts.

Several economists have instead proposed the use of cost-benefit analysis (CBA) [7, 8]. The term CBA is often informally used to refer to any analysis used in decision-making that compares the expected costs and benefits (both in monetary terms) of an investment. In principle, to be regarded as complete, a CBA should capture all benefits due to an intervention, valuing them either at their market value or at the level of consumption that individuals are willing to forego to obtain them. Hence, it has its conceptual roots in welfare economics, which quantifies social welfare in terms of individuals’ willingness-to-pay (WTP) to increase welfare. By using a consistent, directly comparable metric to value all outcomes, CBA allows comparison with non-health interventions. A recent analysis estimated that the return on investment (a form of economic analysis that uses the same economic assumptions as CBA) for vaccines in low- and middle-income countries was comparable or superior to that for non-health interventions such as road safety [3].

The methodology for CEA has been well established, with the perspective or range of costs admissible in a CEA usually prescribed by ‘reference cases’ produced by particular health authorities. In CEAs, the perspective on costs can be narrow (costs and cost offsets falling to healthcare providers alone, as recommended by the National Institute for Health and Care Excellence (NICE) in the UK) [9] or broad (all costs and cost offsets falling on society, as recommended by the World Health Organization (WHO)) [10]. NICE’s recommendation to take a narrow perspective when estimating costs is reasonable given that its evaluations are intended to promote the most efficient use of available resources allocated to the NHS (or publicly funded health sectors) in particular [11]. Conversely, the WHO Guide to Cost-Effectiveness Analysis [10] explicitly recommended all costs and health effects to be valued from the societal perspective, because there are always opportunity costs in every decision we make, such that all costs and resources used for a chosen health intervention (regardless of who paid them) could have been used for other purposes in society, including non-health consumption. The guide further argued that the so-called ‘decision-maker’s approach’ taking such a narrow perspective is not consistent with WHO’s concern that governments should strive to maximise not only the overall health but also wellbeing of societies. The Second Panel on Cost-Effectiveness in Health and Medicine, composed of experts and leaders in the field of health economics, has also provided two reference cases for healthcare sector and societal perspectives, respectively, in their recent report. The Panel recommended that CEAs are undertaken based on both perspectives to improve the quality and comparability of CEAs [5].

Each of these approaches also affects the threshold by which an option with a particular cost-effectiveness ratio is deemed cost-effective. The CEA threshold is often determined based on one of the following: (1) the opportunity cost of new spending at the margin of a budget limit, (2) a multiple of GDP per capita, usually based on human capital arguments (although they have also been justified based on WTP) or (3) preference elicitation (based on WTP) [12]. Using a decision-maker’s perspective and assuming that the decision-maker has control over a budget with the objective of maximising health, the threshold should arguably be set based on the opportunity cost of new spending at the margin of the decision-maker’s budget. From a societal perspective, the threshold should arguably instead be set based on either the human capital value of improved health, or by preference elicitation (based on WTP) of societal willingness to improve health.

In contrast, there is less detail around CBA methodology in health. While there exists guidance on conducting CBAs for government policies [13–15], it generally is not as precise as ‘reference cases’ available for CEAs that specify the exact economic assumptions to be used in pharmacoeconomic evaluations. This is likely because CBA has not been used as extensively as CEA for informing decisions on specific healthcare resource allocation. While CBA is used to evaluate a broader range of public sector initiatives across multiple sectors, CEA guidelines are generally used in the health sector only.

Given the increasing interest in using CBA to evaluate the value of vaccinations and other major public health programmes (as in part evidenced by the Bill & Melinda Gates Foundation’s recent efforts to develop a reference case for CBA), it is important to understand the implications of different approaches for capturing and monetising benefits. To this effect, we conducted a CBA of human papillomavirus (HPV) vaccination as a case study. HPV vaccination is a major public health investment that has been the topic of numerous CEAs with a total of more than 60 studies identified across a number of systematic reviews [16–18]. Indeed, vaccination in general has been subject to numerous studies assessing costs and benefits based on various monetisation methods [3, 19–22]. For the current study, we applied eight different approaches to monetise benefits of HPV vaccination and compared the results.

## Methods

We conducted a CBA of HPV vaccination in the UK. HPV is the aetiological agent of a number of cancers and other diseases such as anogenital warts. Cervical cancer has the highest global burden among the HPV-related fraction of these cancers [23]. In particular, around 70% of cervical cancers are caused by HPV-16 and HPV-18. We have chosen this example as a large public health investment with a well-established model of HPV vaccine impact used for national decision-making, so that our focus in this study could be on the methodology of CBA rather than on the modelling of HPV epidemiology. For simplicity, we focus only on the value of vaccination in preventing cervical cancer due to HPV-16 and HPV-18.

The decision to introduce HPV vaccination in the UK was informed by a CEA that incorporated an epidemiological model of HPV transmission [24] to assess the impact of routine female adolescent two-dose vaccination on cervical cancer burden over a time horizon of 100 years. We adopted the same epidemiological model but used it as input for CBA. We assumed that (1) vaccination is given annually to 12-year-old girls at 80% coverage, with a catch-up campaign in the first year to age 16, and that (2) the vaccine provides lifelong protection against HPV-16 and HPV-18 without cross protection against other HPV types. Costs and benefits were discounted at 3.5% per annum. For the probability sensitivity analysis, we used Latin hypercube sampling to generate 1000 scenarios that encompass the uncertainties in epidemiologic and economic parameters.

The outcome in our CBA was threshold vaccine cost (TVC), which we defined as the maximum vaccine cost per person (including the administration cost) at which HPV vaccination has a benefit-to-cost ratio above one (i.e. the vaccination programme is cost-beneficial) (Additional file 1). The direct benefits of vaccination included all medical cost avoided due to reduced screening for and treatment of cervical cancer and pre-cancerous lesions (Additional file 2: Table S1).

We applied two conceptually different approaches to monetise benefits (lost production and WTP) to examine the impact of varying methods on the results. Estimates of WTP were derived from stated or revealed preference studies while lost production were measured using the human capital and friction cost methods as summarised in Table 1.

**Table 1:** Overview of cost-benefit analysis (CBA) approaches to monetise benefits

Method	Rationale	Major limitations and uncertainties	How we accounted for methodological uncertainties
Human capital	▪ Individual perspective ▪ Indirect benefits are productivity losses avoided by prevented or reduced morbidity and mortality ▪ Productivity losses are valued by individual’s cumulative income over the entire time absent from work	▪ Considers productivity loss incurred only by economically active individuals ▪ Leads to biased decisions in favour of high-income earners and economically active individuals	▪ Sensitivity analysis was conducted to include homemakers in the calculation of productivity loss averted
Friction cost	▪ Employer perspective ▪ Indirect benefits are productivity losses avoided by prevented or reduced morbidity and mortality ▪ Productivity losses are valued by individual’s gross earnings over the friction period ▪ Assumes there always exists some level of involuntary unemployment ▪ The sick/deceased worker is replaced by another worker who otherwise would have remained unemployed	▪ Disease- and job-specific data needed for estimating the friction period are often unavailable ▪ Considers productivity loss incurred only by economically active individuals ▪ Leads to biased decisions in favour of high-income earners and economically active individuals	▪ Sensitivity analysis was conducted to vary friction period – 55, 69 and 90 days – to account for uncertainties regarding the vacancy duration ▪ Sensitivity analysis was conducted to include homemakers in the calculation of productivity loss averted
Value of a statistical life (VSL): Revealed Preference	▪ Individuals implicitly reveal how much they value mortality risk reduction in real markets (e.g. wage-risk trade-offs) ▪ VSL is derived from observed behaviours	▪ Focus is mostly on job-related risks among working-age population, which largely result from injuries rather than illnesses	▪ A range of VSL estimates, including a VSL for cancer after adjusting for a 10-year latency period was used
VSL: Stated Preference	▪ Use contingent valuation with hypothetical scenario (i.e. surveys) to derive VSL	▪ Extra effort may be required to encourage survey participants for valid responses	▪ A range of VSL estimates, including one from a willingness-to-pay (WTP) study done in cervical cancer patients was used
Monetisation of quality adjusted life years (QALYs) (QM)	▪ QALY captures a broad range of health benefits ▪ QALY can be monetised by multiplying the WTP with gains in QALYs	▪ Individuals cannot be expected to have a constant rate of substitution between QALYs and wealth	▪ Our QM approach with a £23,000/QALY WTP is analogous to NICE’s cost-effectiveness reference case, which has a cost-effectiveness threshold of £20,000–£30,000/QALY [9]

### Lost production: Conventional production-based approaches

While conventional CBA generally assumes that individuals are the best judges of their own welfare (i.e. consumer sovereignty) and that monetary values should reflect individual willingness to exchange consumption for the outcomes of concern (e.g. [25], p. 30), lost production has also been commonly used in the CBA literature to value health [26–31]. Under these approaches, productivity loss averted due to reduction in morbidity and mortality were incorporated as indirect benefits of vaccination in terms of the wider economic effects of health as human capital (rather than its intrinsic value).

We considered the two most commonly used production-based approaches, namely the human capital (HC) and friction cost (FC) methods. From the perspective of affected individuals, the HC method assumes that production loss incurred by sick or deceased workers is irreplaceable. The duration of productivity loss for a sick worker was therefore assumed to be the same as the entire duration of disease treatment, whereas productivity loss due to premature death was estimated by assuming an average retirement age of 65. Specifically, production loss was measured with a cumulative sum of income lost over the duration of illness (morbidity) and the number of years lost due to premature death (mortality) using age-specific employment rates and mean personal incomes retrieved from the UK Office for National Statistics [32, 33].

In contrast, the FC method takes the employer perspective and assumes that there always exists some level of involuntary unemployment and hence a sick or deceased worker is replaceable by an otherwise unemployed worker [34]. As such, the FC method only accounts for productivity loss during the friction period, which is defined as the time between the first day of absence of a sick or deceased worker and the last day of training for a replaced worker. According to the 2015 UK Recruitment Trends Report [35] based on responses from major UK recruitment agencies, the average time to fill a vacancy (i.e. time between announcing a job and finding a successful applicant) ranged from 6 to 44 days in 2014. The average time in training for a new employee of 6.8 days was derived from the UK Employer Skills Survey 2015 [36]. As the friction period largely depends on the type of job (e.g. longer friction period for jobs requiring higher-level knowledge and skills) and economic or labour market conditions, it was difficult to find all the necessary data needed to estimate the friction period. We assumed that the sum of (1) the time period between the start of absence by a sick employee and the first day of job announcement and (2) the time period between the acceptance of job offer and the first day of training of a new employee to be approximately 3 to 5 weeks in total based on Koopmanschap’s study [34]. The friction period in the UK was estimated to be approximately 34 to 86 days. In addition to productivity loss incurred over the friction period, we considered additional administrative costs related to hiring (£2610) [36] and training (£5433) [37] a new worker for all mortality and long-term morbidity cases (i.e. treatment time > friction period).

The conventional production-based approaches account for productivity loss from individuals with the paid jobs only and thus disregard homemakers who comprise a substantial proportion of cervical cancer cases (mean age 45, interquartile range 27–59) [38]. As indicated in one of WHO’s guidelines on CBA, the economic value of unpaid work, such as homemaking and caring, is undervalued using this approach [39]. As such, we also considered modified versions of the conventional production-based methods (HC-M and FC-M) in which paid labour and homemakers within the same age group were assumed to have the same economic productivity. The assumption is in line with the UK’s recent effort in recognising the value of unpaid work at home and its contribution to the economy, by providing it with a monetary value equivalent to the average wages of those who are paid to do those tasks [40]. The proportion of homemakers in each age group was approximated based on the Office for National Statistics employment statistics [13].

### Lost production: a new production-based approach

The conventional production-based approach has the advantage that it uses relatively objective and quantifiable measures (e.g. wage rates) compared to a WTP-based approach. However, the theoretical framework of neither the HC nor the FC method is completely sound, because (1) the HC method’s underlying assumption of full employment is often considered unrealistic and (2) the friction period of the FC method largely varies across occupations, times and countries. In order to address both issues, we examined how easily job vacancies could actually be filled within the ‘normal’ friction period in the current UK labour market.

We considered a new approach for estimating productivity loss by interpolating between the HC and FC methods (HC/FC). Under this approach, productivity loss was a weighted average of that under the two methods where the weight for HC corresponded to the proportion of jobs that are unlikely to be filled within the friction period in the current labour market. We estimated this weight based on recent statistics on ‘hard-to-fill vacancies’ (HtFV) from the UK Commission’s Employer Skills Survey 2015 (Additional file 3: Table S2) [36]. HtFV refer to vacancies that are difficult to fill due to skill-related (e.g. lack of qualified applicants) or non-skill-related reasons (e.g. low pay offered for the post). It was noted that there is a major gender difference in occupational employment in the UK [41], with women historically dominating employment in jobs such as leisure and caring while men dominating in construction industry, for example. To take into account the gender difference in occupational employment and largely varying proportions of HtFV by industry sector [36], we calculated the weighted proportion of HtFV for women to be used in the analysis. We compiled two recent UK employment statistics that provide (1) the distribution of female workforce [42] and (2) the proportion of HtFV in 13 industry sectors categorised according to the Standard Industrial Classification [36]. The distribution of females in the workforce largely varied by industry sector, ranging from 0.6% in agriculture to 22% in health and social work, while the proportion of HtFV (regardless of gender) ranged from 23% in education to 43% in construction.

### WTP: the value of a statistical life (VSL) approach

**Fig. 1:**
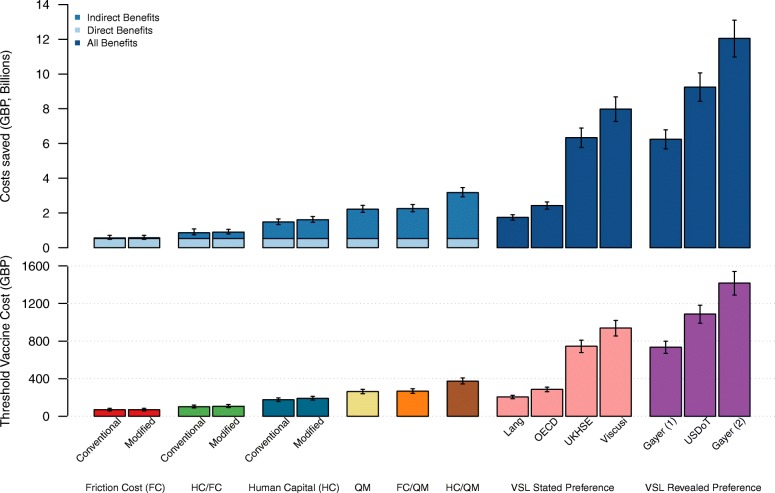
Direct and indirect benefits of two-dose HPV vaccination in the UK (top) and threshold vaccine cost (TVC) estimates (bottom) under different cost-benefit analysis (CBA) approaches

Under this approach, the monetary values of both pecuniary (e.g. avoided medical expenses) and non-pecuniary (e.g. pain and suffering associated with the disease) benefits are presumed to be encapsulated by VSL estimates given that individuals’ WTP takes into account the impact of mortality risk reductions on their wellbeing in every aspect. The VSL estimates are used to value mortality risk reductions and obtained via (1) revealed preferences (VSL-RP) based on labour-market or hedonic wage studies; or (2) stated preferences (VSL-SP) based on contingent valuation studies [8]. While the VSL generally does not address morbidity associated with non-fatal cases, it has been suggested that VSL-RP may as well include the value of the associated morbidity risk though it is likely to be minimal (6–25%) compared to the value of the fatality risk [43]. As for the VSL-SP, there has been mixed evidence regarding the morbidity premium (or cancer premium) to take into account the effects of morbidity associated with the fatality in the VSL estimate [44–46]. This highlights a key advantage of the VSL approach over the HC or FC methods that do not take into account the intrinsic value of health gains. We considered seven different VSL estimates derived from three individual studies (labelled as ‘Lang’, ’Viscusi’ and ‘Gayer 1–2’ in Fig. 1) and three normative national and international guidance (‘UKHSE’, ‘USDoT’ and ‘OECD’) (Additional file 4: Table S3). For VSL-RP, we selected (1) a VSL estimate currently in use by the US Department of Transportation (‘USDoT’) [47], which is very similar to that adopted by the US Department of Health and Human Services [14] and the US Environmental Protection Agency [15], as well as the estimated means from a meta-regression analysis, which adjusted for publication bias [48], and (2) a range of VSL for cancer risk reduction estimated based on hedonic housing prices in the US (‘Gayer 1–2’) [49]. For VSL-SP, we selected VSL estimates from (1) a WTP study conducted among cervical cancer patients in Taiwan (‘Lang’) [50], (2) a recent systematic review of VSL focusing on a ‘cancer premium’ (‘Viscusi’) [45], (3) recommendation of the UK Health and Safety Executive (‘UKHSE’) [13], and (4) OECD guidelines for EU-27 countries (‘OECD’) [44]. All VSL estimates were converted to the current UK currency based on the OECD guideline [44]. To convert VSL values varying across countries and over time to the UK 2015 value, we used the benefit transfer method with income adjustments. For example, to approximate VSL used by the US Department of Transportation, we used purchasing power parity (PPP)-adjusted GDP per capita in the following equation:

$$ {VSL}_{\mathbf{UK},2015}={VSL_{\mathbf{US},2015}}^{\ast}\left( PPP- adjusted\  GDP\  per\ {capita}_{UK,2015}/ PPP- adjusted\  per\ {capita}_{US,2015}\right). $$

Here, PPP-adjusted GDP per capita for both the UK and the US were extracted from the World Bank [51]. To convert the VSL (in USD) estimated from the above equation to the UK currency, we used PPP-adjusted exchange rates from OECD Statistics [52]. To update VSL values across different years (e.g. 2000 to 2015), we used the average Consumer Price Index and Real Income in the UK as follows:

$$ {VSL}_{2015}={VSL_{2000}}^{\ast }\ {\left({CPI}_{2015}/{CPI}_{2000}\right)}^{\ast }\ \left( Real\ {Incomes}_{2014/15}/ Real\ {Incomes}_{1999/00}\right) $$

Data on Consumer Price Index and Real Incomes across different years were available at the UK Office for National Statistics website [53]. After the adjustment, the selected VSL estimates ranged from £1.1 million to £7.2 million. Each adjusted VSL estimate was then multiplied by the projected number of cervical cancer deaths prevented from vaccination.

### WTP: the quality-adjusted life-year (QALY) monetisation (QM) approach

Under this approach, the health outcome in conventional cost-utility analyses, namely QALY, was monetised using individual WTP for an additional QALY gained. Based on a study that assessed WTP for the respondent’s own additional QALY gained (WTP_sel_) in the UK [54], we applied £23,000 to the discounted QALY gained. Our QM approach with a £23,000/QALY WTP is analogous to NICE’s cost-effectiveness reference case, which has a cost-effectiveness threshold of £20,000–£30,000/QALY [9], although our approach is based on individual rather than societal WTP arguments. Hence, it would be expected that the net present value of an intervention using our QM approach would correspond to its net monetary benefit evaluated using NICE’s reference case.

### Integration of production-based and QM approaches

Under these approaches, productivity loss from production-based approaches and monetised QALYs gained were both included when estimating the economic benefit of vaccination (e.g. HC/QM when HC is integrated with QM) to capture both the intrinsic and the instrumental value of better health. Such analyses are analogous to cost-utility analyses using a societal perspective.

Future deaths averted were discounted at 3.5% per annum back to the reference year, i.e. the year in which the vaccination programme is initiated. Subsequently, the value attached to averted mortality was discounted further, depending on the method used. For production-based (HC and FC) and QM approaches, the productivity loss and QALYs lost for each year of life lost due to premature death was discounted back to the year of death. For the VSL-based approaches (VSL-RP and VSL-SP), the same value was ascribed to a prevented death regardless of the age of the woman or the number of life years averted, as has been standard practice for public policy analyses [55].

## Results

Among all CBA methods considered, the WTP-based approach using the VSL yielded the highest TVC estimates. Specifically, the median TVC estimates ranged from £206 (interquartile range: £187–£223) to £939 (£855–£1021) under VSL-SP and £734 (£669–£798) to £1417 (£1291–£1541) under VSL-RP, which correspond to approximately 78.6% and 541% of the TVC estimated under the standard QM method (£262), respectively (Fig. 1). When the QM approach was integrated with the production-based approaches, the TVC estimates ranged from £268 (£244–£293) with FC/QM to £373 (£345–£407) with HC/QM and remained lower than that estimated under the VSL method.

Under the production-based approach, the direct benefit was £0.54 billion (£0.44 billion to £0.66 billion). The mean UK female employment rate used to measure the indirect benefits in terms of averted productivity loss was 36.9% for those aged 16–19 and 64.7–77.6% for those aged 20–64. The indirect benefits varied 9-fold across different monetisation methods utilising the production-based approach, at £33 million in FC, £37 million in FC-M, £946 million in HC, £1.1 billion in HC-M, and £324 million in HC/FC (Fig. 1). Consequently, the FC method resulted in the lowest TVC estimate of £69 (£56–£84), which is only 26% of the TVC estimated under the QM. When integrated with the QM method, the total indirect benefits increased by nearly 53-fold (£1.7 billion) and 2-fold (£2.6 billion) for the FC and HC methods, respectively. Similarly, with homemakers (around 8.9%–13.9% across the different age groups in 2015) included in the calculation of productivity loss under the modified production-based approaches, the TVC estimate increased by 1–2% (from £56–£84 to £57–£85) and 8–10% (from £157–£195 to £172–£211) under the FC and HC method, respectively (Additional file 5: Table S4).

Point estimates and error bars indicate medians and interquartile ranges across 1000 scenarios randomly generated. Benefits under the VSL approaches cannot be decomposed into direct and indirect components. VSL estimates used in Gayer–1 and –2 were derived from the same study using different level of cancer risk [49].

The proportion of HtFV varied by industry sector, ranging from 23% in education (in which 16% of women work) to 43% in construction (in which fewer than 2% of women work). Considering the gender difference and varying proportions of HtFV across different establishments, we estimated that the overall proportion of HtFV among the female workforce in the UK was 31% (Additional file 3: Table S2). That is, we estimated that 69% of all vacancies would be filled with a replaced worker within the friction period. The resulting TVC estimate was £101 (£88–£118) under HC/FC, which was 56% lower and 11% higher than that under HC and FC, respectively. Relative changes in TVC were similar when homemakers were included in the calculation of productivity loss.

We found that the economic benefits of vaccination against HPV-16 and HPV-18 in the UK could vary by as much as 20-fold depending on the method used to monetise benefits. In particular, two-dose HPV vaccination in the UK was found to be not cost-beneficial under the HC approach and all FC-related approaches except when integrated with QM.

## Discussion

Our results suggest that using different approaches to monetise benefits can lead to divergent conclusions about the value of vaccination. Our TVC estimates for a vaccine against cervical cancer in the UK ranged over an order of magnitude (£69–£1417) depending on the method used to value the benefits of cervical cancer prevention. The TVC estimate was lowest (£69–£191) when benefits were valued in terms of productivity loss averted due to ill health and premature mortality, particularly if the friction cost method was used, and highest (£206–£1417) when VSL methodology was used. When an individual WTP for an additional QALY gained was used instead, the TVC estimates (£262–£373) were generally higher than that obtained by valuing productivity gains but lower than that obtained using VSL methods.

Our finding that measuring benefits based on WTP estimates (e.g. the VSL and QM approaches) yields larger benefit estimates than measuring benefits based on lost production (e.g. HC and FC) is unsurprising – this is likely because the former includes both financial (e.g. medical expenses and losses in future earnings) and non-financial (e.g. avoided pain and suffering) benefits of the intervention, whereas the latter solely focuses on lost production [56, 57]. The finding that the FC method yields much smaller benefit estimates than the HC method is likewise intuitive, because the FC method only takes into account temporary losses during the friction period while the HC method assumes lifetime losses during the entire period affected by morbidity and mortality.

Each of the methods used has advantages and limitations. Production-based approaches for valuing health gains have been criticised for not being consistent with the theoretical foundations of CBA in welfare economics, as they focus on changes in productivity rather than measuring overall welfare. Similarly, QALY-based approaches do not fit naturally within the conceptual framework of welfare economics, because they measure changes in health rather than overall welfare. The approach that most directly reflects the principles of welfare economics is to estimate the consumption that affected individuals are willing to trade-off to avoid morbidity or mortality [27, 58, 59].

Valuing benefits based on averted productivity loss has the advantage of being based on an easily measurable quantity (market income). However, for diseases such as cancer, which tend to cause long-term work absences, the difference in outcome between the production-based approaches can be large. In our study, productivity loss estimates under the HC approach were 29 times higher than that under the FC approach. Similarly, Oliva et al. [60] found that the annual productivity cost of mortality due to cervical cancer in Spain was €21.7 million based on HC and €0.39 million with FC (56-fold difference). Advocates of the FC method argue that there is always some level of involuntary unemployment, so the HC method overestimates the societal cost of long-term illness or death by measuring the ‘potential’ productivity loss over the entire period of absenteeism beyond the friction period [34]. The FC method purports to measure the ‘actual’ productivity loss to society from an employer’s perspective by considering the time and related costs (e.g. hiring and training costs) needed to fully restore production levels with a replacement worker.

Both conventional production-based methods have been criticised for valuing life purely in terms of marketable productive capacity and not providing an explicit value for the health gains themselves (i.e. ignoring the additional value of avoided suffering, leisure time and unpaid labour) [61]. The concern is that this may lead to prioritising interventions that primarily benefit high-wage earners over low-wage earners and those doing unpaid labour (e.g. caregiving and housework). In our analysis, we have accounted for unpaid labour by employing the modified versions (namely HC-M and FC-M) and estimate that the TVC for HPV vaccination increases by 22% with the HC and 2% with the FC method if all females are included in productivity loss calculations (data not shown here), rather than those in the paid labour force only. It should be noted, however, that the market value approach that we used measures unpaid household work based on the population average wage, which differs from the conventional method of valuing household based on the average wage of a paid household worker or carer.

Measuring lost production by using wage rates raises a number of methodological questions, including (1) whether or not to assume full employment (we capture this uncertainty by showing results using both HC, which assumes full employment and competitive labour markets, and FC, which does not make these assumptions), (2) whether the economic value of lost productivity is best captured by the employer perspective (so measured in pre-tax wages including fringe benefits and indirect costs) or employee perspective (so measured in post-tax wages), and (3) how to capture labour market constraints on how much work an individual does, since there may be requirements to work a fixed number of hours [62]. For example, Bockstael et al. [63] found that individuals who are required to work a fixed number of hours valued the opportunity cost of time approximately 3.5-fold more than the wage rate, whereas those with flexible working hours valued it similarly to their wage rate.

We proposed an alternative production-based method that may be used instead of established methods, as it strikes a balance between the two approaches in terms of assumptions about unemployment. Weighting the outputs from the two methods according to the proportion of HtFV should theoretically give estimates closer to the actual productivity loss due to ill-health.

An alternative approach is the VSL method. The VSL reflects the marginal rate of substitution between money (or income) and mortality risk and infers the value of the consumption of market goods that individuals are willing to forgo to achieve a reduced risk of premature death [8]. Hence, VSL can be seen as a direct application of the welfarist principle of consumer sovereignty. Consistent with the conceptual framework for CBA, VSL estimates are highly context specific. In practice, however, researchers often rely on the averages across country populations (or even extrapolations from other countries), which can potentially cause under- or overestimations of the result. Furthermore, there are few VSL estimates from low- or middle-income countries. VSL estimates for cancer are particularly divergent, with debates around the existence and magnitude of a ‘cancer premium’ that inflates the VSL for a cancer death in comparison to a death from an acute fatality to incorporate the latency and morbidity period of cancer. For instance, Viscusi et al. [45] suggested the use of 1.21 for cancer premium, the US Environmental Protection Agency, the European Commission and several studies recommend a cancer premium of 1.5 [46, 64, 65], and the UK’s Health and Safety Executive doubles the VSL (or the value of preventing a statistical fatality) estimates of accidental death to derive a VSL estimate for cancer [13]. We understand that there are concerns about transferring VSL between countries with different healthcare systems, income levels and cultural values that may affect mortality risk valuation. Nevertheless, we have used VSL estimates derived from other countries also for the following reasons: (1) there is disagreement and inconsistency with the use of a ‘cancer premium’ when applying a standard VSL to cancer studies and (2) there were few studies reporting cancer-specific VSLs at the time of the study, none of which was from the UK. To minimise such effects, we have adjusted for different income levels and costs between the countries using the ‘unit transfer with income adjustments’ method.

A third approach is to monetise individual WTP for an additional QALY gained. It offers policymakers the flexibility to incorporate additional units for the value of non-health outcomes not captured in measures such as QALYs, as well as to compare outcomes with non-health interventions. In practice, monetised QALYs has been used by government agencies such as the US Department of Health and Human Services [14] and US Food and Drug Administration for regulatory analyses [66]. However, there is still an on-going debate around the use of monetised QALYs in healthcare decision-making among health economists. During the meeting organised by the US National Institutes of Health in 2010, for example, it was argued that QALYs should not be monetised since this approach lacks theoretical and empirical support [67]. It should also be noted that adding productivity costs to monetised QALYs may lead to double counting, as there remains uncertainty about whether productivity loss has been fully captured in QALY measures [5, 6].

Hence, a key challenge to using CBA for priority setting around public health interventions is the great variety in the way benefits can be monetised and the relative lack of detail on normative guidance about the appropriate methodology to use.

## Conclusions

In principle, CBA offers the opportunity to capture many benefits of public health interventions such as vaccination that may not naturally fit into a CEA framework. Other approaches, such as cost-consequences analysis and multiple criteria decision analysis, also admit a wider range of outcomes, but do not offer a straightforward way to synthesise multiple outcomes into a single measure. Wider use of CBA to evaluate public health interventions will require greater convergence around the appropriate methodology to use in order to achieve consistency and comparability across different studies. Ultimately, discussions around appropriate methodology for CBA could help us better understand what we actually value about health.

## Additional files

Additional file 1: Supplementary Text. Summary of the literature review and descriptions on the methodology used for calculating conventional and modified production-based approaches

Additional file 2: **Table S1.** Summary of cost and QALY parameters used in the model

Additional file 3: **Table S2.** Distribution of women in workforce and the proportion of hard-to-fill vacancies across industry sectors

Additional file 4: **Table S3.** List of selected value of a statistical life (VSL) estimates included in the analysis

Additional file 5: **Table S4.** Threshold vaccine cost (TVCs) based on different methods of monetising benefits

## Abbreviations

CBA: cost-benefit analysis
CEA: cost-effectiveness analysis
FC: friction cost
FC-M: modified friction cost method
HC: human capital
HC-M: modified human capital method
HPV: human papillomavirus
HtFV: hard-to-fill vacancies
NICE: National Institute for Health and Care Excellence
PPP: purchasing power parity
QALY: quality-adjusted life-year
QM: monetisation of QALYs
TVC: threshold vaccine cost
VSL: value of a statistical life
VSL-RP: VSL based on revealed preference
VSL-SP: VSL based on stated preference
WHO: World Health Organization
WTP: willingness-to-pay
