# Supplementary material for: Cost-benefit analysis of vaccination: a comparative analysis of eight approaches for valuing changes to mortality and morbidity risks
Source: BMC Med. 2018 Sep 5;16:139. doi: 10.1186/s12916-018-1130-7 (PMC6123970; doi:10.1186/s12916-018-1130-7)
Supplement: Supplementary file 1 — Supplementary Text. Summary of the literature review and descriptions on the methodology used for calculating conventional and modified production-based approaches [file 12916_2018_1130_MOESM1_ESM.pdf]

Additional file 1: Supplementary Text

Literature review: Cost-benefit analysis (CBA) on vaccination

We searched PubMed for articles on cost-benefit analysis of vaccines using the following search query: ("cost-benefit analysis" OR "benefit-cost analysis") AND ("vaccine" OR "vaccination"). The search yielded a total of 269 articles. We narrowed it down to 151 studies by excluding all studies published before January 1, 2000 and further to 135 by excluding articles not written in English. At the initial screening, we reviewed titles and abstracts to exclude (i) cost-effectiveness analysis or any other forms of economic evaluations (i.e. not using monetized benefits), (ii) studies on zoonotic diseases, and (iii) systematic review or comments. At the final stage of screening, we reviewed the full text of 21 remaining articles that conducted a full CBA on vaccination, which includes indirect benefits to see what type of monetization approach was used in the analysis. Only two methods for cost-benefit analysis (CBA) were used in the identified articles, namely the human capital and the value of statistical life approaches. We also found substantial methodological differences among these studies, with the scope of benefits ranging from medical cost savings only, to productivity gains from avoiding disease, to individual or societal willingness to pay to avert premature death. None of these studies examined the implications of varying the approach used to estimate benefits.

Selection of value of statistical life (VSL) estimates from the published literature

To select representative VSL estimates for our analysis, we searched PubMed for articles on a VSL using the following search query: ("value of a statistical life" OR "value of statistical life"), ("revealed preference" OR "stated preference"), ("willingness-to-pay" OR "WTP" AND

"cervical cancer"), and ("hedonic" AND "cancer"). As the stated preference approach using the contingent valuation method is context-specific (i.e. surveys given under the specific hypothetical scenario), we limited our search for WTP studies to that related to cervical cancer only. The search yielded a total of 590 journal articles. After excluding 14 duplicate studies, a total of 576 titles and abstracts were reviewed for relevance based on the following inclusion criteria: (1) the article is written in English; (2) the study reports VSL estimate(s) based on empirical evidences or a systematic review; and (3) the study was conducted in developed countries (comparable to the UK). After the initial screening, we further narrowed it down to include 19 studies that report VSL estimate(s) for cancer, in particular. A full text of the 19 articles that meet the selection criteria were reviewed and the three most relevant studies that provide VSL estimates for cancer were selected to be included in our study. We further added three more VSL estimates from the recent UK and US government reports and OECD's guidelines (based on a systematic review) to see how VSL estimates are applied in practice. A total of 7 VSL estimates including four from relevant studies and three from the national or international normative guidelines were included in the analysis, as shown below. All VSL estimates were converted to the 2015 UK currency using benefit transfer method following the OECD guideline [7].

Estimation of the threshold vaccine cost (TVC)

We defined the TVC as the maximum vaccine cost per person (including the administration cost) at which HPV vaccination has a benefit-to-cost ratio above one (i.e. the vaccination program is cost-beneficial). The TVC estimates presented in Table S4 were computed by dividing the total

benefits (including both the direct and indirect benefits) obtained from each of different CBA methods by the total number of girls to be vaccinated.

References

1. Organisation for Economic Co-operation and Development (OECD): Mortality Risk Valuation in Environment, Health and Transport Policies. 2012. http://www.oecd-ilibrary.org/environment/mortality-risk-valuation-in-environment-health-and-transport-policies_9789264130807-en. Accessed 3 Oct 2016.
