# Supplementary material for: Cost-benefit analysis of vaccination: a comparative analysis of eight approaches for valuing changes to mortality and morbidity risks
Source: BMC Med. 2018 Sep 5;16:139. doi: 10.1186/s12916-018-1130-7 (PMC6123970; doi:10.1186/s12916-018-1130-7)
Supplement: Supplementary file 2 — Table S1. Summary of cost and QALY parameters used in the model [file 12916_2018_1130_MOESM2_ESM.pdf]

Table S1. Summary of cost and QALY parameters used in the model

Parameter	Mean	Distribution
**Treatment/Screening costs**		
Cervical cancer treatment	£16,400	Lognormal (SD: 10,000)
Cytology (liquid-based)	£58	Normal (SD: 15)
Colposcopy	£156	Normal (SD: 39)
Pre-cancerous lesion treatment	£378	Normal (SD: 94)
**Vaccine costs (per dose)**		
Quadrivalent vaccine	£86.50	Fixed (LHS not used)
Administration	£9.33	Fixed (LHS not used)
Vaccine wastage	0.1	Fixed (LHS not used)
**Quality of life weights**		
Cervical cancer treatment	0.285	Triangular (min:0.25, max: 0.32, mode: 0.285)
Post-cancer treatment	0.0305	Triangular (min: 0.00682, max: 0.0542, mode: 0.0305)
**QALY loss per episode**		
Positive cytology result	0.025	Normal (SD: 0.00625)
Positive CIN1 result	0.012	Normal (SD: 0.003)
Positive CIN2 result	0.007	Normal (SD: 0.00175)
Positive CIN3 result	0.054	Normal (SD: 0.00135)
Cervical cancer treatment time (years)	0.116	Lognormal (SD: 0.36)

Source: Jit M, Brisson M, Laprise JF, Choi YH. Comparison of two dose and three dose human papillomavirus vaccine schedules: cost effectiveness analysis based on transmission model. BMJ 2015; 350: g7584.
