# Supplementary material for: Cost-benefit analysis of vaccination: a comparative analysis of eight approaches for valuing changes to mortality and morbidity risks
Source: BMC Med. 2018 Sep 5;16:139. doi: 10.1186/s12916-018-1130-7 (PMC6123970; doi:10.1186/s12916-018-1130-7)
Supplement: Supplementary file 3 — Table S2. Distribution of women in workforce and the proportion of hard-to-fill vacancies across industry sectors [file 12916_2018_1130_MOESM3_ESM.pdf]

Table S2. Distribution of women in workforce and the proportion of hard-to-fill vacancies (HtFV) across industry sectors

**Industry**	**Women (%)**[1]	**HtFV (%)**[2]
Agriculture	0.6	30
Manufacturing	5.8	37.5
Construction	1.8	43
Wholesale	13.2	30
Accommodation/Food Services	6.0	33
Transport and Communication	4.7	37
Financial Services	3.8	23
Business Services	12.3	33
Public Administration	6.3	27
Education	15.9	23
Health/Social Work	22.1	35
Arts and Other	6.6	39

Source:

1. Office for National Statistics (ONS). EMP04: Employment by occupation. 2016. Available from: https://www.ons.gov.uk/employmentandlabourmarket/peopleinwork/employmentandemployeetypes/datasets/employmentbyoccupationemp04
2. UK Commission for Employment and Skills (UKCES). The UK Commission's Employer Skills Survey 2015: UK Results, 2016.
