# Supplementary material for: Cost-benefit analysis of vaccination: a comparative analysis of eight approaches for valuing changes to mortality and morbidity risks
Source: BMC Med. 2018 Sep 5;16:139. doi: 10.1186/s12916-018-1130-7 (PMC6123970; doi:10.1186/s12916-018-1130-7)
Supplement: Supplementary file 4 — Table S3. List of selected value of a statistical life (VSL) estimates included in the analysis [file 12916_2018_1130_MOESM4_ESM.pdf]

Table S3. List of selected VSL estimates included in the analysis

Approach	Source	VSL estimate
Revealed Preference (VSL-RP)	Gayer et al. (2002)[1]	£3.75 million
	US Department of Transportation (2015)[2]	£5.46 million
	Gayer et al. (2002)[1]	£7.24 million
Stated Preference (VSL-SP)	Lang et al. (2014)[3]	£1.05 million
	OECD (2012) [4]	£1.46 million
	UK Health and Safety Executive (2000)[5]	£3.80 million
	Viscusi et al. (2014)[6]	£6.49 million

Source:

1. Gayer T, Hamilton JT, Viscusi WK. The market value of reducing cancer risk: Hedonic housing prices with changing information. Southern Economic Journal 2002; (69): 266-89.
2. U.S. Department of Transportation. Revised Departmental Guidance on Valuation of a Statistical Life in Economic Analysis. In: U.S. Department of Transportation, editor.; 2016.
3. Lang HC, Chang K, Ying YH. Quality of life, treatments, and patients' willingness to pay for a complete remission of cervical cancer in Taiwan. Health Econ 2012; 21(10): 1217-33.
4. OECD. Mortality Risk Valuation in Environment, Health and Transport Policies, OECD Publishing; 2012. http://dx.doi.org/10.1787/9789264130807-en
5. UK HM Treasury. The Green Book: Appraisal and Evaluation in Central Government. In: HM Treasury, editor. London, UK; 2003.
6. Viscusi WK, Huber J, Bell J. Assessing whether there is a cancer premium for the value of a statistical life. Health Econ 2014; 23(4): 384-96.
