# Supplementary material for: Cost-benefit analysis of vaccination: a comparative analysis of eight approaches for valuing changes to mortality and morbidity risks
Source: BMC Med. 2018 Sep 5;16:139. doi: 10.1186/s12916-018-1130-7 (PMC6123970; doi:10.1186/s12916-018-1130-7)
Supplement: Supplementary file 5 — Table S4. Threshold vaccine cost (TVCs) based on different methods of monetising benefits [file 12916_2018_1130_MOESM5_ESM.pdf]

**Table S4. TVCs based on different methods of monetizing benefits**

| Approach | | TVC (Median, £) | Interquartile Ranges (£) |
|---|---|---|---|
| FC | Employed | 68.5 | (56.0, 84.1) |
| FC-M | Employed + Homemakers | 69.1 | (56.5, 84.7) |
| FC/HC | Employed | 101.3 | (87.9, 118.5) |
| FC/HC-M | Employed + Homemakers | 106.5 | (92.6, 124.3) |
| HC | Employed | 175.0 | (157.4, 194.6) |
| HC-M | Employed + Homemakers | 190.9 | (171.5, 211.2) |
| VSL-SP | Lang et al, 2014 | 205.6 | (187.2, 223.5) |
| QM | £23K/QALY | 262.4 | (239.7, 287.0) |
| FC/QM | Employed \| £23K/QALY | 267.5 | (244.0, 293.1) |
| VSL-SP | OECD (EU-27 countries), 2012 | 286.0 | (260.4, 310.9) |
| HC/QM | Employed \| £23K/QALY | 373.1 | (344.8, 406.7) |
| VSL-RP | Gayer et al, 2002 (min) | 734.3 | (668.6, 798.2) |
| VSL-SP | UK Health and Safety Executive | 744.9 | (678.3, 809.7) |
| VSL-SP | Viscusi et al, 2014 | 939.1 | (855.1, 1020.8) |
| VSL-RP | US Department of Transportation | 1087.9 | (990.6, 1182.6) |
| VSL-RP | Gayer et al, 2002 (max) | 1417.0 | (1291.0, 1541.0) |
